# Parental perspectives on the decision-making process on transport mode choice in adolescents: a qualitative study with mothers and fathers

**DOI:** 10.3389/fpsyg.2023.1227612

**Published:** 2023-09-14

**Authors:** Clara Tristram, Anne K. Reimers, Denise Renninger, Franziska Beck, Yolanda Demetriou, Isabel Marzi

**Affiliations:** ^1^Department of Sport Science and Sport, Friedrich-Alexander-Universität Erlangen-Nürnberg, Erlangen, Germany; ^2^Department of Sport and Health Sciences, Technical University of Munich, Munich, Germany

**Keywords:** decision-making process, Thematic Analysis, active travel, physical activity, parents, youth, family

## Abstract

**Objective:**

The present study aims to understand the familial decision-making process on transport mode choice in adolescents with a focus on the parental perspective within this process.

**Background:**

Active travel contributes to adolescents’ overall physical activity and its positive health effects. Based on the social-learning theory, especially parents are assigned a central role for adolescents’ travel behavior. The aim of the present study was to examine how parents are involved in the decision-making process on transport mode choice in adolescents.

**Method:**

The study is part of the cross-sectional mixed-methods ARRIVE study which includes semi-structured interviews with mothers (*n* = 12) and fathers (*n* = 7) of 11- to 14-year-old German adolescents. The interviews focused on travel behavior in adolescents and the decision-making process on transport mode choice from the parental perspective. All interviews were analyzed inductively using Thematic Analysis.

**Results:**

Our study revealed that parents do not primarily decide for or against active travel in adolescents, but are mostly involved in the decision-making process, especially in case of a deviation from the main transport mode. Different forms of parental involvement in the decision-making process were identified. Some parents acted as main decision makers which is the highest form of involvement while others gave their children complete freedom of choose a transport mode for themselves. These parents accepted their child’s choice fully which shows a low involvement in the decision-making process.

**Conclusion:**

The results provide a deeper understanding of the familial decision-making process on travel behavior in adolescents. The results indicate an occasionally parental involvement in the decision-making process on the mainly used transport mode by adolescents, and that mothers and fathers are always involved when deviating from the main mode.

**Implications:**

Further research should investigate changes in travel behavior from childhood to young adulthood to understand long-term travel decisions in families. Due to the findings that parents are often involved in the decision-making process on transport mode choice and that they mainly reported safety concerns as barriers to their children’s active travel, further research should focus especially on the social and physical environment of adolescents.

## Introduction

In public health, the importance of active travel in everyday life for the promotion of physical activity and health in youth as well as the environmental benefits of active travel are widely discussed (Larouche et al., 2014; Yang et al., 2014; Brand et al., 2021). Current studies indicate that daily active travel, such as walking or cycling, has positive effects on mental and physical health, especially in youth (Gordon-Larsen et al., 2005; Larouche et al., 2014).

Despite these benefits, the number of adolescents who travel actively to school is low worldwide (Grize et al., 2010; Fyhri et al., 2011; Haug et al., 2021; Reimers et al., 2021). Additionally, a study from New Zealand that took data from 2014, 2016 and 2018 into account shows that active travel has steadily decreased in children and adolescents over the years (Smith et al., 2019). In Scotland, 42.1% of adolescents aged 11, 13, and 15 years walked and only 1.1% cycled both ways to and from school in 2018 (Haug et al., 2021). In Germany, less than 50% of adolescents aged 11 to 17 years walked or cycled to school (Reimers et al., 2021). Although the prevalence of active travel to school is relatively high in Germany compared to other countries, there is still room for improvements to promote physical activity and thereby health in adolescents. To increase physical activity in youth by considering the choice of transport mode, a change from passive transport modes to active travel is reasonable. Therefore, it is obligatory to understand how the decision-making process on transport mode choice in adolescents occurs within families.

In this context, the term ‘decision-making process’, as we define it, refers to the specific process that parents and adolescents go through when talking about transport mode choice. Thereby, ‘decision-making’ can either be a conscious process which is accompanied by verbal communication, or an unconscious process without any verbal exchange between parents and adolescents (Kremers et al., 2006). To our understanding, ‘decision-making process’ describes the procedure of weighing up the positive and negative sides of different transport modes by taking various influences into account (e.g., psychosocial and environmental influences) and ends with a determination for a specific transport mode.

According to the social learning theory by Bandura and Walters (1963) indicating learning processes can be caused by observing the behavior of human role models. Considering these findings in relation to travel behavior, it does not seem unreasonable that parental travel behavior, their attitudes and norms toward it as well as parenting styles and practices may influence adolescent’s travel behavior (Mâsse et al., 2017).

Additionally, a current study indicates that cognitive autonomy support from parents, which can be seen as similar to providing choice, positively predict adolescents’ intrinsic values and self-efficacy (Zimmermann et al., 2021). Regarding transport mode choice, it can be assumed that autonomy support from parents, and thereby the provision of choice, seems to have a positive effect on adolescents’ self-confidence. Therefore, transport mode choice in adolescents may be dependent on the extent to which parents enable their children or further support them in making their own decisions.

Family research in general shows that the relationship between parents and their children changes during adolescence (Ecarius, 2007). While the parenting style in childhood is predominantly characterized by obedience and subordination, these values change toward different parental goals in adolescence, such as more independence and space for the child’s free will (Ecarius, 2007). In family research, adolescence is seen as the phase with the greatest number of conflicts between parents and children, so parents are looking for more compromises through negotiations with their child. Adolescents, on the other hand, strive for more autonomy and independence from their parents (Ecarius, 2007). Based on this knowledge, it can be concluded that the changing parent–child-relationship during adolescence might has an impact on decision-making processes within families, including those on transport mode choice in adolescents.

Current research offers a number of quantitative studies identifying parental barriers to adolescents’ active travel to school (Aranda-Balboa et al., 2020). These barriers include perceived distance to the intended destination, perception of crime and traffic-related safety, the design of the built and social environment, such as ‘walkability’, the accompaniment of peers (Bungum et al., 2009), time issues, convenience, physical and motivational barriers, or concerns about the weather (Ahlport et al., 2008; D'Haese et al., 2011; Napier et al., 2011; Chillon et al., 2014). Perceived distance and safety issues are the most stated parental barriers to active travel to school (Babey et al., 2009; D'Haese et al., 2011). Although these quantitative studies show that parental attitudes and perceptions are associated with active travel in adolescents, they often focus only on the factors promoting and barriers hindering active travel and do not consider the underlying mechanisms and the decision-making process that occur within the family before deciding on a specific transport mode (Aranda-Balboa et al., 2020).

Further, current research provides only a few qualitative studies examining the role of parents for transport mode choice in adolescents (Ahern et al., 2017; Pelletier et al., 2021). Apart from commonly identified barriers to active travel in quantitative studies, qualitative research identifies further parental barriers to and promoters of active travel in youth, such as perceived social norms, perceived change of the idea of parenting through generations, and the appraisal of adolescents’ competence (Ahern et al., 2017; Forsberg et al., 2020). Furthermore, Crawford, Bennetts (Crawford et al., 2017) identify differences in the parental and child’s perspective on transport mode choice in a sample of children, adolescents and parents, mainly mothers (Crawford et al., 2017). In their study, children and adolescents reported a wider range of safety concerns but had generally a more positive attitude toward independent and active mobility than parents. In addition, the study indicates that children and adolescents seek to influence their parent’s opinion on different transport modes by, for example, citing the amount of peers’ freedom on independent mobility as reference points when discussing with them (Crawford et al., 2017).

While qualitative studies show that the parental perspective on adolescent’s decision-making process on transport mode choice is influenced by different barriers to and facilitators of active travel, other aspects that are important for understanding the familial decision-making on transport mode choice are missing. First, most of the existing studies consider solely the way to school but neglect ways to other destinations, such as friend’s houses, shops, or leisure facilities (Ahern et al., 2017; Forsberg et al., 2020). It seems important to take the ways to non-school destinations into account, since the way to school is obligatory while non-school ways are mostly made voluntarily. Additionally, a recent study on transport mode choice in adolescents reveals that transport mode choice to school does not necessarily reflect transport mode choice to non-school destinations (Marzi et al., n.d.). Further, ways to non-school destinations might be influenced by other factors than the way to school as, for school ways, parents choose commonly the easiest and quickest option for reasons of time (Faulkner et al., 2010). Second, previous studies focus mainly on children by disregarding adolescents and their interaction with parents on transport mode (Forsberg et al., 2020). Considering older children could yield new insights, since previous evidence reveals that the child’s age influences parental concerns about active travel (Johansson et al., 2012; Huertas-Delgado et al., 2017). Last, research on travel behavior not sufficiently involve the maternal and paternal perspective on the decision-making process on transport mode choice in adolescents (Ahern et al., 2017). A differentiated consideration of the maternal and paternal perspective seems to be beneficial, since the mother’s and father’s role within the family is associated with diverse allocations (Baxter and Smart, 2010). For example, mothers tend to be more often in charge of adolescents by planning their daily appointments while fathers spent more time at work (Hobler et al., 2017; Hipp and Bünning, 2020).

In summary, more research is needed to get a deeper understanding of parental involvement in adolescents’ decision-making process on transport mode choice and a holistic view on parental experiences during this process.

## Theoretical background

Panter et al. (2008) provide a ‘Conceptual Framework for the Environmental Determinants of Active Travel in Children’ as the basis for the relationship between environmental as well as individual factors and active travel in youth by examining existing literature on transport mode choice. The developed theoretical framework includes various determinants and their interrelationship that may influence the decision-making process on transport mode choice in adolescents, such as physical environmental factors (e.g., infrastructure), parental and adolescents’ perception of the social and physical environment, and their individual factors (e.g., socio-demographics and attitudes). The framework also shows that parental perceptions of the social and physical environment as well as mothers’ and fathers’ attitude toward different transport modes have a decisive influence on the decision-making on transport mode choice in adolescents. In general, parents’ personal attitude on healthy lifestyle has an impact on their children’s health behavior (Niermann et al., 2018).

Based on the research gaps identified as well as theoretical framing stated above, the present study aims to examine how parents are involved in the decision-making process on transport mode choice in adolescents and how the decision on transport mode occurs within the family. Therefore, we conducted semi-structured interviews with parents and chose an exclusively inductive approach for analyzing the data (Reimers et al., 2022). The present study does not only consider the adolescents’ way to school but also ways to non-school destinations, such as friends’ homes or leisure facilities.

## Methods

### Study design

The current study is part of the mixed-methods cross-sectional ARRIVE study (Active tRavel behavioR in the famIly enVironmEnt) that aimed to examine travel behavior in adolescents and its predictors (Reimers et al., 2022). The current analysis is based on the qualitative part of the ARRIVE study that includes semi-structured interviews with families to develop a deeper understanding of the decision-making process relevant to adolescents’ transport mode choice. Parents and adolescents were interviewed separately, however the current study considers only the parental interviews to focus on mothers’ and fathers’ perspective on the decision-making process. Ethical approval for the study was received from the ethics commission of the Friedrich-Alexander-Universität Erlangen-Nürnberg, Germany (Reg. 249_21 B).

### Participants and recruitment

For the study, 12 families were recruited using theoretical sampling methods (Nagl-Cupal, 2013). The sample was designed with respect to socioeconomic status, migration background, sex/gender, and environmental conditions of the families. Further, all families were recruited through personal contacts of the research team. Because of one additional interview with an adolescent, altogether 13 adolescents aged 11 to 14 years (7 male and 6 female), 12 mothers (aged 44 ± 3.3 years) and 7 fathers (aged 44 ± 3.4 years) were interviewed. All parents were employed and lived in different degrees of urbanization in Germany, such as rural areas, small towns, medium-sized towns and cities.

### Data collection

The collection of data took place between September and November 2021. Before collecting the data, interviewers were trained from an interview expert and sample interviews were conducted (Reimers et al., 2022). After participants gave their written consent to take part in the study, they received an individual link for an online meeting. All interviews were conducted via the online tool Zoom (zoom.us). The online setup enabled the participants to complete the interview wherever they wanted. Before the interview started, participants were informed about the procedure and got the possibility to ask questions. They were also allowed to refuse answering any question during the interview. After clarifying all formalities, the interview and the recording of audio files started. At the end of the interview, the recording was stopped and saved. All interviews were conducted in accordance to the interview guideline. The interviews lasted between 11 and 38 min. After the interviews, socio-demographic characteristics (e.g., age, employment, degree of urbanization) of the family members were assessed via a short questionnaire.

### Interview guideline

The semi-structured interviews focused on the adolescents’ travel behavior and the decision-making process that occurs within the family before deciding on a transport mode. A ‘decision tree’ was used to structure the interviews (Reimers et al., 2022). Further, the ‘Conceptual Framework for the Environmental Determinants of Active Travel in Children’ by Panter et al. (2008) served as a basis for the interview guideline. At the start of the interview, each parent was instructed to report on any trip their child had made during the last week. This trip was either an active or a passive one. After that, they were asked to remember a trip to the same destination where the adolescent had used a different transport mode (active/passive). This procedure was repeated for another destination. To get a deeper understanding of the influences on the decision-making process, parents were asked to talk about their experiences during the situation at home (e.g., before the adolescent traveling to school or other destinations) and their child’s experience during the trip itself (e.g., weather conditions, type of transport mode). At the end of each interview, parents were asked which type of transport mode they preferred for adolescent’s travel in general. Due to uncertainties in the first interviews, we changed the order of the topics in the second versions of the guideline to enable a better interview start. Additionally, we changed the option between active vs. passive transport modes to ever occurring changes in transport modes to the addressed destination (‘Have you ever used another transport mode to XY, for example cycling instead of walking, or the car instead of the bus?’) to identify differences in mobility behavior not only between passive and active but also between different transport modes. More detailed information on the interview guideline can be found elsewhere (Reimers et al., 2022).

### Data analysis

All interviews were transcribed verbatim using a guideline according to Dresing and Pehl (2018) and checked by two researchers (DR, IM). For the transcription of the interview audios and the data analysis, the software transcribe the interviews and code and analyse the videos^1^ was used. Data were analyzed using an inductive form of Thematic Analysis (Braun and Clarke, 2006, 2012). This method offers a qualitative approach for data collected in more applied research and helps to identify, analyze and report patterns within these data. It also organizes and describes the hole data set in great detail (Braun and Clarke, 2006). The aim of Thematic Analysis is to find patterns in qualitative data without forming a theory and present them in a way that is understandable for everyone (Braun and Clarke, 2006, 2014). The analysis consists of six steps, which are not separated from each other by strict borders but merge into each other. The six steps are (1) familiarizing yourself with the data, (2) generating initial codes, (3) searching for themes, (4) reviewing the themes, (5) defining and naming the themes and (6) writing a report (Braun and Clarke, 2012). Since Panter et al.’s framework (Panter et al., 2008) provides an general overview of various determinants of active travel in children and adolescents but does not specify the individual determinants in detail, we deliberately opted for an inductive approach to get deeper insights on the decision-making process on transport mode choice in adolescents. Therefore, the outcomes presented in the following section are the result of an exclusively inductive approach. More information on the methodological approach can be found elsewhere (Reimers et al., 2022).

## Results

The results of the present study show that the parental involvement in the decision-making process on transport mode choice in adolescents differed between the decision on the mainly used transport mode and in case a deviation occurred, both for the way to school and ways to non-school destinations. As mentioned above, all results presented in the following are gained through an inductive approach to allow for deeper insights in the decision-making process on transport mode choice.

First, we identified parental non-involvement as well as involvement for the mainly used transport mode and deviations from it in the decision-making process:

1. Parental non-involvement
2. Parental involvement – Decision on main transport mode
3. Parental involvement – Deviation from main transport mode

Second, we identified various degrees within the parental involvements:

1. Parental involvement – Decision on main transport mode
  - Main decision maker
  - Excluding specific transport modes
  - Role modeling
  - Encouragement
  - Accepting
2. Parental involvement – Deviation from main transport mode
  - Main decision maker
  - Making an offer
  - Material support

In the following, a detailed description of the decision-making process on adolescent’s mainly used transport mode is considered first, followed by a presentation of the results on the decision-making process for deviations from the main transport mode. Afterwards, differences between the maternal and paternal perspective on transport mode choice are presented. The following presentation of the results includes more sequences from interviews with mothers as we interviewed more mothers due to single-parent families (only single mothers).

### Parental non-involvement

In our interviews, some mothers and fathers reported that they did not know which transport mode adolescents used, that they did not care about it, or that they would not force adolescents to use a specific one. This shows that these parents were not directly involved in the adolescents’ decision-making process because they left the decision completely to the adolescent. Here, parents did not influence their child by giving advices or forcing it to use the transport mode preferred by them:

“R: But then I leave that up to him [which transport mode he prefers].

I: There you let him decide then, how he likes to/

R: // Yes //

I: / wants to drive. All right.”

*(Mother of son F9, trip to school, bus)*

“Yes, I think she likes [riding the bike] quite a bit, so probably better than taking the tram, otherwise she would not do that. So we do not force her to take specific transport modes.”

(Father of daughter F12, trip to school, tram)

“It does not happen that often [that he walks to the shop] but yeah I think most of the time he decides [which transport mode he wants to use] himself. So that is actually where I say / He takes his scooter or his bike or he walks so that is his own decision.”

(Mother of son F13, trip to shop, walking)

Another degree of parental non-involvement showed up in situations where adolescents did not even have the choice between different transport modes, and had to take the only one that was possible for them due to various environmental conditions. For example, some families lived in a rural area with long distances to various destinations, did not own a car or had no time to drive, and furthermore the walking and cycling paths were insufficient. Due to these environmental circumstances, the adolescent’s only option was to take the bus to school:

“I: How did it come to the decision that [son] goes to school by bus?

R: I do not think he had any other choice because I did not have time to drive him and my wife did not have a driver's license at that time and the route to school is fastest by bus.”

(Father of son F3, trip to school, bus)

In this interview it can be seen that the decision-making process on transport mode choice for the way to school was not characterized by parental involvement but restricted through environmental conditions. Here, the father presented himself in a very passive position, without the intention to interfere in his son’s decision. He placed the responsibility for arriving at school on his son, who had to make the decision. Even though the father could enable more options by driving his son with the car, his passive viewpoint limited his son to the only option of taking the bus. Regarding the ‘Conceptual Framework’ (Panter et al., 2008) it can be seen that the environmental factors stand in distant connection with the parental characteristics and attitudes. Therefore, our finding that these environmental factors appear in parental non-involvement on the decision-making process stay in line with the theoretical assumptions.

### Parental involvement – decision on main transport mode

**Figure 1:**
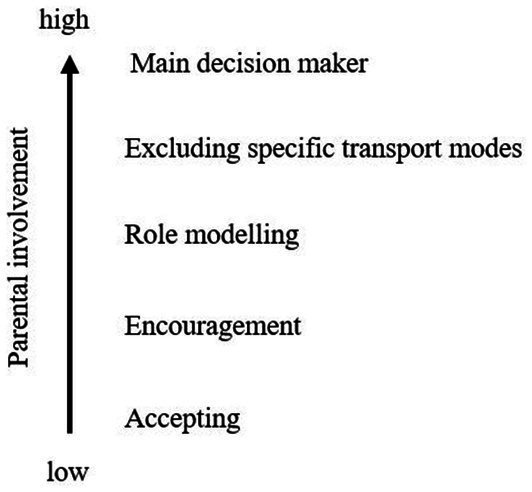
Decision on main transport mode. Identified degrees of parental involvement in the decision-making process on adolescents’ main transport mode (own illustration based on the Thematic Analysis).

In addition to parental non-involvement in the decision-making process, our results show that a larger proportion of parents was involved in the process. In most of these cases, adolescents were allowed to choose their preferred transport mode but their mothers and fathers took part in the previous decision-making process and influenced adolescents by different degrees. It should be noted here that one degree of involvement always referred to one specific transport mode. Therefore, we found that the parental involvement differed not only between the decision on the mainly used transport mode by adolescents and deviations from it, but also between different transport modes. Therefore, it occasionally happened that we identified more than one degree of involvement within one interview. Figure 1 shows all identified degrees, arranged from low to high parental involvement in the decision-making process on transport mode choice.

Parents acting as main decision makers as well as the exclusion of active and passive transport modes, and thereby limitation of available transport options for adolescents, shows a high parental influence on the decision-making process because parents do not only give advice but already make the final decision on the used transport mode or exclude specific ones without involving or asking their child.

The degrees ‘role modeling’, ‘encouragement’ as well as ‘accepting’ show a low parental involvement in the decision-making process on transport mode choice because the final decision on the adolescent’s mainly used transport mode lies with the young people. Although parental involvement is evident in all decisions on the main transport mode, the various identified degrees show higher and lower levels of parental involvement in the decision-making process.

#### Main decision maker

Our analysis revealed that some parents decided directly which transport mode their child had to use by relieving adolescents of their decision. This parental decision was mostly associated with passive mobility. Parents indicated that they chauffeured their son or daughter by car, without asking the adolescent about his/her choice on transport mode. With regard to the way to school, parental reasons for acting as main decision makers were usually organizational aspects. For example, some reported that they got more time in the morning when driving their daughter or son to school. In relation to ways to non-school destinations, parents usually decided to chauffeur adolescents because they perceived the distance to the intended destination as too long for using an active mode (e.g., walking was preferred for distances up to 1 km, cycling for distances up to 5 km and taking the car for distances over 5 km), and simultaneously the public transport infrastructure as insufficient, or they had concerns about their child’s safety, especially when it was dark outside:

“I: We already said [earlier in the interview] if it is agreed beforehand like that / You practically decide together [with other parents] that he rides the bike?

R: Exactly. Actually, these are like carpools that the parents have, like WhatsApp groups. And then we discuss 'The weather is nice, the boys/Could go by bike now.' But of course that also means in return, when you pick them [the children] up again in the evening, a driver with a luggage rack must be available to take the two big bikes and the boys back/

I: That is, you rather make the decision then how he drives?

R: Yes, it's usually like that, yes. [...] That was more clear in the summer, [that] he also came back home by bike. And now in winter we [parents] decide that and say, 'Today the weather is good, today you go there by bike and we pick you up'.”

(Mother of son F9, trip to leisure activity, bike)

In this interview, it can be seen that there were differences between the seasons. In summer, it was more self-evident for the adolescent to take the bike to leisure activities. In winter, the mother talked to other parents about the transport mode they considered most suitable for their children. Therefore, this decision depended on the social environment as well as organizational aspects of the family, for example forming carpools as seen in the interview sequence above, and was usually made spontaneously. In both situations, summer and winter, the parents were the main decision makers on the adolescent’s used transport mode.

Another situation from which parents emerged as main decision makers were ways to be traveled together. In these cases, parents determined their personally preferred transport mode to be used:

“I: Do you remember who actually made the decision that you [mother and daughter] would go there together by bicycle and not otherwise?

R: Well, if it means we are going somewhere together, then it is actually clear that we are going by bike. So unless it is so explicitly further ways, then we go by car.

I: Ok. But otherwise, if it is feasible by bike and you are traveling together/

R: Yes then we actually always go by bike exactly.”

(Mother of daughter F12, ways to other destinations, bike)

#### Excluding specific transport modes

Another degree of involvement is the exclusion of specific transport modes. Here, parents limited adolescents’ transport mode options, either by excluding passive transport modes, such as the car, or active ones, such as cycling. Some parents refused to drive their children due to time issues or because they wanted the adolescent to gain independence. Furthermore, some mothers and fathers reported that they did not have time to drive adolescents to and from places, or are strictly against a full-time ‘parental chauffeuring service’. Most of these parents did not have any concerns about their child’s personal safety and supported the use of active transport modes, mostly the bike:

“So in the summer, I think it is also a bit more normal that he rode his bike. We [parents and son] just said that if he starts a second sport where he is so active, he has to accept that he takes over his logistics partly himself, because we just cannot drive him somewhere twice every day.”

(Mother of son F9, trip to leisure activity, bike)

In this interview, it becomes clear that the mother linked their son’s decision for/against starting a new hobby with a condition which related to travel behavior. If the son would start an additional leisure activity, then he had to manage the way to it by himself and travel independently. The mother justified her position by her limited time resources. Further, it can be seen that due to the maternal behavior, the choice of transport mode had a direct influence on the organization of their son’s leisure time.

Additionally, parents decided against parental chauffeuring as they did not travel by car themselves:

“I: Then I would be interested to know how it came that [son] walks to school regularly?

R: It actually already developed in elementary school, so he always walked to elementary school on his own. We started doing that little by little, and because we very seldom go to work by car, we did not really have the option of saying that he would go by car. With the bicycle I think it is not necessarily meaningful. So I do not see now that he would drive with the bicycle that/There was never an option and there was also actually never a protest from his side.

I: So he also supported the decision?

R: Exactly, so in elementary school he wanted to walk on his own early in the morning anyway. We then simply passed that on to the secondary school. Exactly.”

(Mother of son F13, trip to school, walking)

Besides the exclusion of passive transport modes, situations occurred where parents excluded active transport modes, for example cycling, because they estimated the cycling infrastructure as dangerous and were concerned about their child’s safety in road traffic. The following example shows that a mother prohibited using the bike to school because of a combination of safety concerns and bad occurrences she had heard of. Here, the mother did not ask her son about his preferred transport mode but excluded the bike:

“I do not think there was any question of him riding his bike, because it was simply like that/At that time it was still hundred on the country road, which is also very narrow. And where you already/Also there is already someone crashed and run over. So in no case now, I would have left my fifth grader drive there.”

(Mother of son F9, trip to school, bus)

#### Role modeling

Another degree of parental involvement in the adolescents’ decision-making process on their mainly used transport mode is parental role modeling reflected by mother’s and father’s personal attitude toward active travel and physical activity in general. In the interviews, some parents reported that they raised adolescents in such a way that active travel became a matter of course for them. Additionally, many parents mentioned that physical activity plays an important role in their daily life and that they regularly travel actively by themselves, for example to get to work or leisure activities. Most of these physically active parents wanted to pass their positive experiences on their children. Thus, the decision on the used transport mode often depended on the maternal and paternal preference and how parents raised adolescents. Within this degree, mothers and fathers acted as role models for their daughter or son since they had the intention to get their child into more physical activity through active travel:

“R: Well, I think it is very important that they [adolescents] also take shorter routes/Or certain routes that are easy to walk and where I say that the time is feasible without any problems. These ways are without time pressure. That you can just say/We also go sometimes into the city for example. So that is about a quarter of an hour from here. Because you sit around all day anyway. My husband and I both have an office job and the children are at school all day, and I think it is extremely important to say that you do not always just get in the car and drive the short distance, but you also walk sometimes. So if it goes to the shops or also times around, I personally find this important. Because you move far too little. So I run a quarter of an hour at work and I also like to do that and I also notice when I go by car, on these days I miss that [walking] too.

I: So this is also a behavior that you would like to pass on to your children?

R: Exactly, that is why they do not get a ride to school.”

(Mother of son F13, trip to school and ways to other destinations, walking)

In this interview, the mother set an example of an active lifestyle for her children through walking to destinations instead of using passive transport modes, such as the car. She had the intention that her children perceived and imitated this active lifestyle. Due to the fact that she wanted her children to imitate her travel behavior, she further excluded parental chauffeuring to school and other destinations.

Parental attitude toward physical activity played an important role in this degree. In our interviews, some mothers and fathers described the importance of physical activity in their personal daily life and that they tried to teach adolescents the relevance of physical activity for health through active travel. Here, parents acted as role models for adolescents because they had the intention of demonstrating an active lifestyle.

Additionally, in the interview below it becomes clear that the father was raised to be independent himself. This is why teaching his son independence is important to him, also with regard to travel behavior. He intended to raise his son the same way he was raised by his parents – learning to travel actively and independently:

“Yes, it is just a pity, but because they [adolescents] only become independent later, yes. The [son], he does not know it any other way. And I know that from myself, in the past, my parents all went to work very early and I had to get up myself, I had to make my own food, I had to go out myself, lock up, and in the afternoon, when I came, I was also alone. That was just the way it was and you were independent much faster than when you are taken to everything and everything is organized and you just have to take care of yourself.”

(Father of son F13, trip to school, walking)

#### Encouragement

Another form of parental involvement in the decision-making process is ‘encouragement’. In this case, mothers and fathers left the decision on the used transport mode completely to their child without forcing him/her to use a parental preferred one. Furthermore, they endorsed the adolescents’ decision, and thereby encouraged them in using active or passive transport modes. In some of these situations, it turned out that adolescents chose a passive transport mode which their parents supported. The following sequence shows that the father supported his daughter in using the bus to school:

“I: How do you feel about the bus ride? So that [daughter] now regularly takes the bus?

R: I think it is good. That is because I have the background that I use over more than 15 years [public transport] myself […] And I actually feel that public transportation is quite efficient because the school bus goes directly to the school which means that it would be much more time-consuming, much more energy-consuming if we [the parents] had to drive them [the children] as ‘helicopter parents’. No that is actually important to us that [daughter] makes this way to school relatively independently, and that is where the bus comes in.”

(Father of daughter F11, trip to school, bus)

Moreover, some parents reported that they did not have any concerns about adolescents using an active transport mode and that they would leave their son walking alone since primary school. An example for this can be seen in the following sequence where the father reported that he and his wife supported the use of an active transport mode for the way to school:

“I: And what do you think about [son] walking to school every day?

R: The best thing ever. I have always had that myself, so I know how it is to walk the same way to school for years. I [walked to] elementary school to ninth grade and only from the ninth I then went by bus and that was never really nice. Because you are always on the bus for ages. A school bus works pretty cool here, it is an established system, but you still sit on the bus for an hour every day and that is just/We [the family] do not have a problem here [with the walking infrastructure]. [With walking] it is ten minutes [to school] and ten minutes back.”

(Father of son F13, trip to school, walking)

#### Accepting

Within this degree, parents did not fully agree with the adolescent’s decision on its preferred transport mode. Although adolescents were free to decide on their mainly used transport mode, parents did not actively support their child’s decision but just accepted it. Mothers and fathers did not prohibit the chosen transport mode and accepted their child’s choice even if they had concerns about it:

“I: And if you think about the way now, this way to the bus stop, then you would rather prefer that he walks, that he rides a scooter or that he is brought?

R: So in principle that they [son and his brother] walk. That is the way it was meant to be. The scooter has just turned out that they like to ride it and use it from time to time.”

(Mother of son F2, trip to school, bus)

In this interview, it can be seen that the mother declared walking to the bus station as the norm. She did not like the idea of her sons using the scooter to the bus station but also did not prohibit it. She agreed to this transport mode as long as its use remained an exception and her children generally continued walking to the bus.

In further interviews, mothers and fathers suggested the use of an active transport mode (e.g., bike) but the adolescent opted for a passive one (e.g., bus). Here, parents did not force their preferred transport mode on their daughter but accepted her decision on taking the bus to school:

“I: And how do you feel about [daughter] taking the bus and choosing to do that?

R: Well, I think it would be nicer if she actually rode a bike, the older sister actually does everything by bike now, because she just wants to have independence and also during the breaks at school/I mean in eleventh grade there are often free hours during the day. [The sister] is then just mobile with the bike and I think it would be nicer if [daughter] rides a bike too. Because I [know it from myself], I also went to school by bike and think that by riding the bike, you build up an incredibly good condition from which, I would say, [you benefit your] whole life. But she [daughter] cannot be convinced. Not even in the summer, of riding a bike.

I: Okay, that would be my next question, what [daughter] attitude is towards taking the bus, or towards not riding a bike?

R: I assume that she is more comfortable, it is not so exhausting [taking the bus]. She is rather a bit lazy in everyday life I would say.”

(Mother of daughter F11, trip to school, bus)

In this interview, it can be seen that the adolescent’s choice on transport mode did not agree with the parental choice. Even if the father did not share the same opinion as his daughter, he still let her decide which transport mode she wanted to use and did not interfere further in the decision-making process but accepted the chosen mode.

We identified different reasons for parental disagreement. One reason was the parents’ positive attitude toward physical activity. For example, one mother suggested that her daughter should walk at least one way to town actively but at the same time accepted her decision not to walk and take the bus for the way there and back instead:

“What I think is good for example/My suggestion was always, ‘Why don't you walk a route and drive one then with the bus?’ as an example. Such a combination, but as I said with the walking, they [daughter and her friends] are not so enthusiastic.”

(Mother of daughter F7, trip to town, bus)

In the interview shown above, the mother tried to convince her daughter to use an active transport mode, but her daughter decided to use the bus (passive mode) for the way to town and back home. Even if the mother preferred her daughter to walk at least one way, she accepted her daughter’s decision on using the bus for both ways. In this interview, the mother reported that she uses mostly the car for her daily traveling. Further, she described herself and her family as ‘non-cyclists’. Therefore, it can be seen that the mother tried to get her daughter into active travel although she predominantly uses passive transport modes herself.

### Parental involvement – deviation from main transport mode

**Figure 2:**
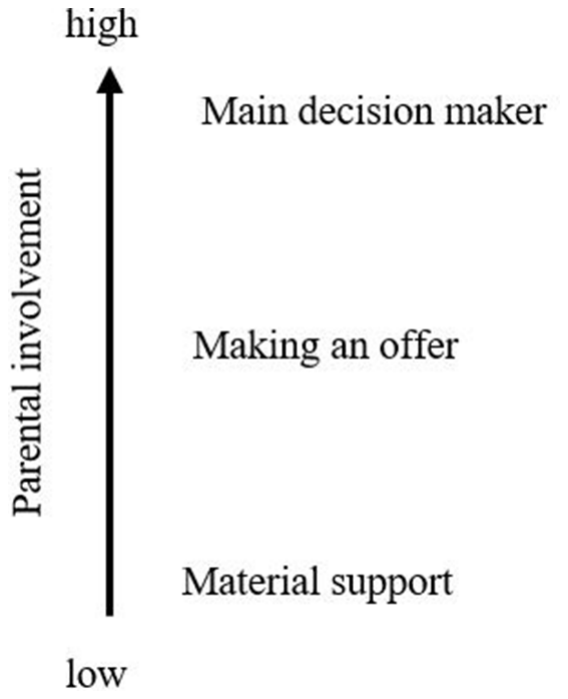
Deviation from main transport mode. Identified degrees of parental involvement in the decision-making process on deviations from adolescents’ main transport mode (own illustration based on the Thematic Analysis).

In situations where adolescents used another transport mode than usual, our results show that parents were always involved in the decision-making process on transport mode choice. Figure 2 shows all degrees, arranged from low to high parental involvement.

#### Main decision maker

Similar to decisions on main transport modes, the analysis of the interviews revealed that parents were also in cases of deviations from the mainly used transport modes involved as main decision makers. For example, some parents kept their children from using an active transport mode (walking or cycling) due to bad weather conditions (e.g., heavy rain or snow). In situations where parents acted as main decision makers, adolescents did not always agree with their mother’s or father’s choice on transport mode but had to accept it and use the parental preferred one. In the following interview, the mother decided to chauffeur her son to school by car due to heavy rain:

“I: Is [son] then the one who asks 'Can we go by car today?' or from whom does the decision then come?

R: No, rather from us parents, so we say, ‘We are going to do it [drive with the car] now’.

I: And does [son] is being asked [if he wants to be driven]?

R: No. That is determined.

I: And is he then always satisfied with the decision or does he then say no he would rather walk?

R: No, that fits. Or sometimes there are discussions, so if it is about power, you cannot discuss everything. We determine that and he sees it then also mostly exactly yes.”

(Mother of son F13, trip to school, car)

The interview above shows that the mother was not even willing to discuss possible transport modes, and the associated advantages and disadvantages, with her son. To her, it was perfectly fine to determine the transport mode used by her son regardless of his wishes and needs.

Besides parental chauffeuring, some parents did not decide against an active and for a passive transport mode but replaced an active mode (e.g., cycling) with another active one (e.g., walking). We found different reasons of parental displacement of transport modes, for example bad weather conditions:

“He prefers to ride his bike. I do then rather intervene and say he has to walk when the weather is bad. When it rains, when it snows, I prefer him to walk. Simply so that the school things do not get wet / There are now accidents when it gets dark again in the morning […] Ok he does not leave until half past seven, because then I would also rather him walk than ride his bike.”

(Mother of son F1, trip to school, walking)

Additionally, parents reported that they decided to drive their child to school due to oversleeping:

“I: And your daughter told me that you asked her just a short time ago [to drive her to school] and she did not know exactly why. She could not tell me whether it was because of the weather, or?

R: There was, no, that was really recently, there was exactly the situation when one of them [daughter and her friends] had to go to school / [They did not know the time] when they wanted to meet, and then she was on a phone call with one friend, then I said, ‘Now is quarter you have to go, I will drive you [to school] now. [...] You cannot spend another quarter of an hour discussing when you are leaving the house.’

I: Ok and then you also made the decision?

R: Then I said, ‘So we are leaving now’. And I think, exactly, she was then ok with it.”

(Mother of daughter F7, trip to school, car)

Another reason for parental main decision-making was safety issues for example due to darkness:

“So now they [son and his friends] sometimes have training in a gym. That is in the evening. Then we do not want them to still be riding their bikes around at seven in the evening. Then we drive them, too. Then we also do a carpool like that.”

(Mother of son F13, trip to leisure activity, car)

In all of these cases, the adolescent’s decision-making process on transport mode choice was determined by parents and they were kept from the opportunity to decide by themselves which transport mode they wanted to use.

#### Making an offer

Due to various circumstances, such as bad weather, combining ways or much luggage, some mothers and fathers offered adolescents a ride in the car. In this degree called ‘making an offer’ it was not the adolescent who asked for parental chauffeuring but mothers and fathers offered him/her a ‘parental chauffeuring service’. Adolescents were then free to accept or decline the parental offer. In most of these situations, adolescents usually used an active transport mode but parents offered them a passive one:

“So as I said, if he has extra luggage with him, if just the weather is extremely bad. I have already offered him / There was already a thunderstorm coming up / There was thunderstorm predicted, just at noon when school would close. So if at noon, when the school is out, and a thunderstorm is going on there. Then he should call me, then I will pick him up.”

(Mother of son F1, trip to school, car)

Besides situations where adolescents accepted the parental offer, we also found situations where adolescents declined the offer. In the following, the father offered his son a drive to school due to the fact that he drove his younger son anyway but the adolescent did not always accepted this offer and sometimes used his main transport mode instead:

“Yes, when it is raining dogs and cats outside, he [son] is happy when I [drive his brother and take him in the car], because […] then he likes that he can just jump out [of the car], but he does not need that. So sometimes it gets on his nerves and then he says, ‘Go without me’ if he has to wait for his brother when [his brother is too slow], then he just says, ‘I am going now’, because it is just not far.”

(Father of son F13, trip to school, walking)

Here, the father offered his son a ride in the car due to bad weather conditions. Normally, the son was happy to be driven when it rained heavily. On the other hand, he sometimes declined his father’s offer when he had to wait for his little brother. Then, the adolescent preferred to use his main transport mode (walking) for the way to school instead.

#### Material support

With this degree, parents supported adolescents materially in form of parental chauffeuring to destinations on adolescents’ request. Situations where parents supported adolescents materially always arouse by the young people asking their parents for chauffeuring, and mothers and fathers in turn mostly complied with their child’s wish:

“I: And then you make the decision that the car is better or does the [son] comes to you?

R: Half half.

I: That means?

R: Yes he [asks me that] he would [like] to be driven/ [He asks] whether I can drive him and then I do not have a choice.”

(Father of son F3, trip to school, car)

The most common reason for adolescents asking for chauffeuring was heavy rain:

“So if it is raining cats and dogs, then he already comes and says, 'Mommy drive me’, but that is, as I said earlier, as an exception sometimes absolutely okay.”

(Mother of son F10, ways to other destinations, car)

In the interviews above, it can be seen that adolescents asked their parents for material support in exceptional situations, such as bad weather conditions. Most of the parents complied with the adolescent’s request for chauffeuring due to the fact that taking the car was always an exception.

### Differences between mothers’ and fathers’ perspective

Besides parental non-involvement and involvement as well as the various degrees of involvement in the decision-making process, the present study focused further on differences between the maternal and paternal perspective on the decision-making process on transport mode choice in adolescents. Although in most cases mothers and fathers had similar opinions about their child’s used transport mode, we also found situations in which parents had different perspectives on transport mode choice and behaved differently in the decision-making process. In the following sequence, the mother reported that she would not drive her son to school in case of oversleeping

“I: //But you would rather not do that [drive him to school when he is late] //

R: That would depend on the rest of the situation. If it was on the way to kindergarten, and I was driving anyway, then of course I would take him with me. But if I had planned my day different, then I would say, 'No, I am sorry, you have to ride the bike.' That has also happened.

I: That he is also a bit responsible for himself?

R: Yes, exactly.

I: All right. And your husband [would drive him?]

R: Yes. More ‘mothering’ I would say.”

(Mother of son F9, trip to school, car)

while the father said that he would prefer driving over being late for school:

“So I have to admit, to school, I almost never have to [drive him], because he always gets the bus. But otherwise my opinion is that if he misses the bus, it can still happen, it can also happen with us adults, I do not want to leave him standing there in the rain and he is late for school. That is always unpleasant. And for me it is, of course, unfavorable, but I am just flexible with my working time and can just drive the detour. And then I prefer this over him being late.” (Father of son F9, trip to school, car)

Here, it can be seen that the mother behaved stricter than the father. While the mother said that her son had to manage traveling by himself in case of being late, the father reported that he would offer his son a ride in the car to safe him from embarrassing situations. Here, mother and father had different perspectives on how to handle exceptional situations. In terms of parental involvement the example reflects that the mother avoided being involved (non-involvement) while the father acted in form of ‘making an offer’.

Additionally, some parents had different opinions on the transport modes adolescents were allowed to use. Therefore, these parents made oppositional decisions and were either not involved in the decision-making process (‘I do not really care’, mother) or excluded a specific transport mode (forbade to use the scooter, father) with regard to their child’s transport mode choice. The following example shows that the mother allowed using the scooter for the way to the bus station

“Well, I do not really care. I can understand that they [two brothers] also want to go by scooter, because they still have a way to walk. The bus stop is not in front of the house, [and they] have to go down the hill here / So they have to go down the hill and also back up in the afternoon // That is why // [...] I can imagine it being nicer with a scooter.” (Mother of son F2, trip to bus station, scooter)

while the father had forbidden, and therefore excluded, using this transport mode:

“So I had actually forbidden to drive with the scooter.”

(Father of son F2, trip to bus station, walking)

Even if the father had forbidden his sons to use the scooter, they finally took this transport mode to the bus station.

The most common difference between mothers and fathers was the responsibility for the children. We found that mothers seem to be more in charge of the children than fathers (e.g., by shaping their daily routine or having their daily appointments in mind) because fathers were often busier with work:

“I: Can you tell me a little bit about how the decision was made for him to go there by bike and not any other way?

R: Yes. So the decision is rather from us [parents] and I think also in agreement with the other parents. [...] We did that by the fact that I am not always there when they went to field hockey, my wife has organized that mainly and there is a WhatsApp chat, mainly from mothers.”

(Father of son F9, trip to leisure activity, bike)

“I: I would be interested, or that you describe to me again, what happens in the morning in your family until [son] is actually on the way [to school]?

R: I cannot tell much about that, because mostly, either I am already at work or I am still in bed, because I only came back from work at three in the morning.”

(Father of son F3, trip to school, bus)

In the second interview above, the father reported clearly that he “cannot tell much” about the family’s morning routine because he is mostly busy with his work. This shows that the responsibility for his son’s daily routine and appointments, and thereby also mobility routines, lay with his wife.

While the father can be assigned to parental non-involvement for the way to school, the mother of this family excluded driving her son to school:

“I: And how did it come that [son] always takes the bus? So how was this decision made?

R: Yes [the distance to school] is a bit far for walking and I think it is unnecessary to drive him. First of all, I do not have the time at all and secondly, he can easily take the bus. I also took the bus to school when I was a kid.

I: And do you know if [son] is happy with this decision [of taking the bus to school]?

B: I think mostly he is. But he also would not mind if we drove him there and back regularly I guess.”

(Mother of son F3, trip to school, bus)

With respect to our theoretical background, although it becomes clear that the ‘Conceptual Framework for the Environmental Determinants of Active Travel in Children’ (Panter et al., 2008) takes physical environmental factors as well as individual factors, including parental characteristics, attributes and perceptions of the environment into account. However, it does not specify the parental factors and their part regarding the decision-making process on transport mode choice in adolescents. The results stated above complement the framework by separating parental involvement and non-involvement and looking at differences between the mothers’ and fathers’ perspective.

## Discussion

The aim of the present study was to examine how parents are involved in the decision-making process on transport mode choice in adolescents and how the decision on transport mode occurs within the family. Our results show that parents did not necessarily make the decision on adolescents’ transport modes but were mostly involved in the decision-making process on transport mode choice, both for the decision on the main transport mode and situation-specific deviations from it. This parental involvement was shaped by different degrees.

Although the determinant *decision-making process* is part of the ‘Conceptual Framework for the Environmental Determinants of Active Travel in Children’ (Panter et al., 2008) which serves as the basis for the present study, it is not explained in more detail and gives no information about the influence that parents, adolescents and children have in the travel mode choice process.To the best of our knowledge, this is the first study that focused on transport mode choice in adolescents from the parental perspective that occurs before the final decision for and against certain transport modes is made while many previous studies have focused predominantly on younger children (Forsberg et al., 2020; Pelletier et al., 2021) and on barriers to active travel (Wilson et al., 2018; Aranda-Balboa et al., 2020).

Our results indicate that only in a few interviews, parents were not involved in the decision-making process on transport mode choice. In cases of non-involvement, parents did not know or care about the transport mode adolescents used and did not force them to use a specific one. If parents were not involved in the process, they enabled adolescents to make their own decisions and learn to organize traveling by themselves (e.g., planning time for traveling with different transport modes, inquiring about bus connections, etc.). Thereby, these parents did not only promote adolescents’ active but also independent mobility that has significantly decreased worldwide due to increased safety concerns of parents (Francis et al., 2017; Marzi and Reimers, 2018) and an increase in motorization (Blinkert, 2004).

With regard to the decision on the main transport mode, both for the way to school and ways to non-school destinations, some parents acted as main decision makers by deciding on adolescents’ transport mode. Due to various barriers, such as safety concerns (e.g., missing sidewalks or darkness) or bad weather conditions (e.g., heavy rain or snow), the most parental chosen transport mode was the car. Similarly, previous studies have indicated safety concerns as parental barrier to active travel but focused predominantly on children (D'Haese et al., 2011; Napier et al., 2011; Francis et al., 2017). For example, in the Netherlands, more people use the bike for their daily trips than in Germany which is associated with a better infrastructure for cycle paths (Pucher and Buehler, 2008). Due to this, increasing walking and cycling infrastructure in Germany may help to reduce parental safety concerns and therefore support active transport in adolescents.

By deciding for parental chauffeuring, parents kept adolescents from choosing an (active) mode by themselves. Here, mothers and fathers reduced adolescents’ opportunities of being physically active and limited thereby the positive health outcomes associated with active travel (Larouche et al., 2014).

Furthermore, our results show that parents were highly involved in the decision-making process by excluding specific transport modes, which was mostly the car. In the interviews, parents highlighted that they wanted their children to gain independence and responsibility and thus, strictly avoided a ‘parental chauffeuring service’. Additionally, as the present study focused predominantly on adolescents, parental safety concerns inducing car travel seemed to be less apparent than in children (Forman et al., 2008). Furthermore, we interviewed some families from rural areas, where overall parental safety concerns are lower than in cities (Lopes et al., 2014). In cases where parents excluded parental chauffeuring as a passive form of travel, the adolescent was left to decide itself on its preferred transport mode and probably chose an active transport mode instead of a passive one which further promotes an active lifestyle (Yang et al., 2014).

Another degree of parental involvement in the decision-making process was ‘role modeling’. The finding that parental mobility behavior played an important role in the decision-making process stays in line with previous studies indicating that the family environment and thereby parent’s behavior-specific characteristics have an influence on children’s and adolescents’ behavior (Jacobi et al., 2011; Niermann et al., 2018). For example, positive associations between parental and adolescents’ mobility behavior have been found (Carlson et al., 2014). Furthermore, our finding stays in line with the social learning theory according Bandura and Walters (1963) which states that learning processes can be caused by observing the behavior of human role models. Due to the theory, it is not surprising that physically active parents act as role models for adolescents and follow the intention to set an example of an active lifestyle, also in relation to mobility behavior. Additionally, parental mobility behavior and parents’ attitude toward active travel may influence parenting styles. It could be concluded that the decision for and against various transport modes does not occur spontaneously but is based on mobility routines that have been developed within the family over several years.

Our analysis revealed that many mothers and fathers supported and encouraged adolescents in the decision on their main transport mode. In these situations, adolescents chose mainly an active mode, such as walking or cycling, and their parents encouraged them in using these active modes. Previous studies indicated that social support in general, and parental support specifically, has an influence on adolescent’s physical activity level (Cheng et al., 2014; Niermann et al., 2018). Adolescents who receive higher social support, especially from their parents, show higher levels of physical activity (Reimers et al., 2019). Furthermore, adolescents who get strong family support are more likely to choose an active transport mode for the way to and from school (Loureiro et al., 2022). These findings highlight the importance of parental support for the promotion of active travel.

In addition, our results show that parents were usually even more involved in case of a deviation from the main transport mode than when deciding on the main mode. A deviation from the mainly used transport mode occurred predominantly due to unusual events, such as exceptional weather conditions (e.g., heavy rain or snow), spontaneous appointments or other commitments within the family. In all analyzed interview situations, the car replaced the usual transport mode. Thus, adolescents transport mode changed mostly from an active to a passive one. At first sight, this change from an active to a passive transport mode seems to have negative consequences on adolescent’s physical activity level. Using a passive transport mode, for example driving with the car, deprives adolescents of an opportunity to achieve the 60 min of moderate-to-vigorous physical activity per day recommended by the World Health Organization (WHO, 2020). However, at second sight, it becomes apparent that deviations from the main transport mode used by adolescents are rather rare because of the parental reasons stated for the deviation, which were predominantly bad weather conditions. Thus, it remains unclear how these irregularly occurring deviations impact adolescents’ active travel and overall physical activity level.

Finally, we found that mothers and fathers were to different degrees involved in the decision-making process on transport mode choice in adolescents. Some fathers reported that their wife had the main responsibility for the children in the family. This goes hand in hand with the fact that these fathers were usually less informed about adolescents’ travel behaviors and less involved in the decision-making process. An explanation for this can be found in the different roles that mothers and fathers have within families. It seems as if fathers are still more responsible for earning money and therefore often busier with their work, while mothers take care of the children: In Germany fathers are still more likely to have a full-time job while mothers work mostly part-time (Hobler et al., 2017; Hipp and Bünning, 2020). Additionally, in most societies men earn more money than their female counterparts (Bundesministerium für Familie, Senioren, Frauen und Jugend, 2010; Boll and Lagemann, 2019). Because of this salary imbalance between men and women, and of social stereotypes that push most women in the role of the primary caregiver for children, mothers are more in charge of structuring children’s and adolescent’s daily routines and appointments than fathers (Baxter and Smart, 2010) which is also linked to travel behaviors. Furthermore, maternal, but not paternal, walking for transport is positively associated with higher walking in children (Schoeppe et al., 2017). Due to this and the fact that they may be more concerned about their children’s safety, mothers are probably more involved in the decision-making process. Possible differences between the mothers’ and fathers’ perspective for their child’s decision on transport mode, as we found them here, have received little attention in previous studies (Ahern et al., 2017; Ross et al., 2018). For example the ‘Conceptual Framework’ (Panter et al., 2008) does include parental determinants regarding adolescents’ active travel but does not separate between mothers and fathers. Therefore, further research should take a closer look on differences between mothers and fathers as well as their influence on adolescent’s decision-making on transport mode choice.

## Implications

The aim of the present study was to examine how parents are involved in the decision-making process on transport mode choice in adolescents. The results show that parents were occasionally involved in the process when deciding on the mainly used transport mode, and that to different degrees. When adolescents deviated from the main mode, for example due to bad weather conditions or limited time resources, mothers and fathers always made the decision on the used transport mode. Here, they mostly preferred driving adolescents in the car. Although deviations from the main transport mode and thereby a parental-induced switch to passive mobility only occurred irregularly, it is still important to establish an active mobility behavior in the whole family in order to reduce the number of passive trips in everyday life.

Additionally, more research on the social (e.g., peers or siblings) and environmental factors (e.g., crime or traffic related safety concerns) is required as we found that most of the barriers to active travel in adolescents related to the physical and social environment.

Moreover, future research should examine how travel behavior changes from childhood to young adulthood in order to understand long-term travel decisions in the family and be able to explain travel behavior in adolescents. This seems even more important since our results, which show that parents act as role models for their children to develop an active lifestyle, and thereby active mobility behavior, are in line with previous studies (Jacobi et al., 2011; Niermann et al., 2018).

Additionally, parental need-supportive behavior may be a key factor in understanding how travel behavior changes from childhood to young adulthood since current studies indicate that different classifications of need-supportive and motivational behaviors serve as basis for intervention studies (Teixeira et al., 2020; Ahmadi et al., 2022).

In general, future interventions that aim to promote active travel should not be limited to children and adolescents but need to include parents since they are involved in the decision-making process on transport mode choice to different degrees, especially when there are deviations from the main transport mode.

## Strengths and limitations

A major strength of the present study is its focus on parental perspectives on the decision-making process on transport mode choice in adolescents while many previous studies have concentrated mostly on younger children (Crawford et al., 2017; Francis et al., 2017; Forsberg et al., 2020). Further, the sampling procedure was designed with respect to age, sex/gender, living area and different types of ways, such as the way to school or ways to non-school destinations. Especially the intended destination can influence transport mode choice due to a recent study that shows that transport mode choice for the way to school does not automatically reflect the choice for ways to non-school destinations (Marzi et al., n.d.). Within the sampling, we paid attention to a balanced selection of families first consisting of mother, father and at least one adolescent, and second consisting of mother and adolescent only. Finally, the study includes both the perspective of mothers and of fathers by interviewing them separately. In addition, the present study features some limitations. One of them is the under-representation of fathers. Altogether, we interviewed 12 mothers and 7 fathers which shows an imbalance between the sexes. This unequal distribution results from the fact that some families were single-parent households and always single mothers. Another limitation is that the survey of the interviews took place during the Covid-19 pandemic. Thus, restrictions related to the pandemic may have influenced the families travel behavior.

Strengths and limitations can also be found in the area of the selected research methods. First, a major strength of the semi-structured interviews may be its possibility to get deeper and more detailed information from participants during the interview process. A limitation may be that a smaller sample can be collected than, for example, when using survey questionnaires (Mey and Mruck, 2010). Second, the chosen Thematic Analysis offers a high flexibility which can be seen as a major strength of this method. Simultaneously, this flexibility can also lead to inconsistencies and lack of coherence in the development of themes derived from the research data (Holloway and Todres, 2003). Furthermore, it cannot be excluded that the present qualitative study may be a subject to interpretation bias of the coder, despite efforts to adhere to the validation of the coding framework.

## Conclusion

The present study provides new insights into the decision-making process on transport mode choice in adolescents, by taking interviews with mothers and fathers of adolescents aged 11–14 years from Germany into account. The results indicate an occasionally parental involvement in the decision-making process on the mainly used transport mode by adolescents, and that mothers and fathers are always involved when deviating from the main mode. In both cases, the degrees of parental involvement varied. Due to the finding that parents were often involved in the process, and that especially their upbringing associated with their own travel behavior influenced adolescents’ decision-making, further research should focus on the adolescents’ social environment. Therefore, future research should consider the physical environment of adolescents which also seems to influence parental perceptions of safe traveling and thereby their involvement in adolescents’ travel behavior.

## Data availability statement

The raw data supporting the conclusions of this article will be made available by the authors, without undue reservation.

## Ethics statement

The studies involving humans were approved by Friedrich-Alexander-Universität Erlangen-Nürnberg, Germany (Reg. 249_21 B). The studies were conducted in accordance with the local legislation and institutional requirements. Written informed consent for participation in this study was provided by the participants’ legal guardians/next of kin.

## Author contributions

AR, DR, FB, IM, and YD designed the ARRIVE study. CT and IM conceptualized the current investigation. CT analyzed the data and wrote the original draft of the manuscript. AR, DR, and IM reviewed the manuscripts throughout the writing process. AR and IM supervised the current investigation. All authors read and approved the final manuscript.

## Funding

We acknowledge financial support by Deutsche Forschungsgemeinschaft and Friedrich-Alexander-Universität Erlangen-Nürnberg within the funding programme “Open Access Publication Funding”.

## Conflict of interest

The authors declare that the research was conducted in the absence of any commercial or financial relationships that could be construed as a potential conflict of interest.

## Publisher’s note

All claims expressed in this article are solely those of the authors and do not necessarily represent those of their affiliated organizations, or those of the publisher, the editors and the reviewers. Any product that may be evaluated in this article, or claim that may be made by its manufacturer, is not guaranteed or endorsed by the publisher.

## Footnotes

^1^ audiotranskription.de

## References

[ref1] Ahern S. M. Arnott B. Chatterton T. de Nazelle A. Kellar I. McEachan R. R. C. (2017). Understanding parents' school travel choices: a qualitative study using the theoretical domains framework. J. Transp. Health 4, 278–293. doi: 10.1016/j.jth.2016.11.001

[ref2] Ahlport K. N. Linnan L. Vaughn A. Evenson K. R. Ward D. S. (2008). Barriers to and facilitators of walking and bicycling to school: formative results from the non-motorized travel study. Health Educ. Behav. 35, 221–244. doi: 10.1177/1090198106288794, PMID: 18094097

[ref3] Ahmadi A. Noetel M. Parker P. Ryan R. Ntoumanis N. Reeve J.. (2022). A classification system for teachers’ motivational behaviours recommended in self-determination theory interventions. J. Educ. Psychol. doi: 10.31234/osf.io/4vrym

[ref4] Aranda-Balboa M. J. Huertas-Delgado F. J. Herrador-Colmenero M. Cardon G. Chillón P. (2020). Parental barriers to active transport to school: a systematic review. Int. J. Public Health 65, 87–98. doi: 10.1007/s00038-019-01313-1, PMID: 31728600

[ref5] Babey S. H. Hastert T. A. Huang W. Brown E. R. (2009). Sociodemographic, family, and environmental factors associated with active commuting to school among US adolescents. J. Public Health Policy 30, S203–S220. doi: 10.1057/jphp.2008.61 19190574

[ref6] Bandura A Walters RH. Social learning and personality development. Holt Rinehart and Winston: New York; (1963).

[ref7] Baxter J Smart D. Fathering in Australia among couple families with young children, Canberra: Australian Government Department of Families, Housing, Community Services and Indigenous Affairs (2010).

[ref8] Blinkert B. (2004). Quality of the city for children: chaos and order. Child. Youth Environ. 14, 99–112. doi: 10.1353/cye.2004.0064

[ref9] Boll C. Lagemann A. (2019). The gender pay gap in EU countries — new evidence based on EU-SES 2014 data. Intereconomics 54, 101–105. doi: 10.1007/s10272-019-0802-7

[ref10] Brand C. Dons E. Anaya-Boig E. Avila-Palencia I. Clark A. de Nazelle A.. (2021). The climate change mitigation effects of daily active travel in cities. Transp. Res. Part D: Transp. Environ. 93:102764. doi: 10.1016/j.trd.2021.102764

[ref11] Braun V. Clarke V. (2006). Using thematic analysis in psychology. Qual. Res. Psychol. 3, 77–101. doi: 10.1191/1478088706qp063oa

[ref12] Braun V. Clarke V. (2012). “Thematic analysis” in APA handbook of research methods in psychology, Vol 2: Research designs: Quantitative, qualitative, neuropsychological, and biological. eds. Cooper H. Camic P. M. Long D. L. Panter A. T. Rindskopf D. Sher K. J. (Washington: American Psychological Association)

[ref13] Braun V. Clarke V. (2014). What can “thematic analysis” offer health and wellbeing researchers? Int. J. Qual. Stud. Health Well-being 9:26152. doi: 10.3402/qhw.v9.26152, PMID: 25326092 PMC4201665

[ref14] Bundesministerium für Familie, Senioren, Frauen und Jugend. Entgeltungleichheit zwischen Frauen und Männern. Einstellungen, Erfahrungen und Forderungen der Bevölkrung zum “gender pay gap”. (2010).

[ref15] Bungum T. J. Lounsbery M. Moonie S. Gast J. (2009). Prevalence and correlates of walking and biking to school among adolescents. J. Community Health 34, 129–134. doi: 10.1007/s10900-008-9135-3, PMID: 18931894

[ref16] Carlson J. A. Sallis J. F. Kerr J. Conway T. L. Cain K. Frank L. D.. (2014). Built environment characteristics and parent active transportation are associated with active travel to school in youth age 12–15. Br. J. Sports Med. 48, 1634–1639. doi: 10.1136/bjsports-2013-093101, PMID: 24659503 PMC4447304

[ref17] Cheng L. A. Mendonca G. Farias Junior J. C. (2014). Physical activity in adolescents: analysis of the social influence of parents and friends. J. Pediatr. 90, 35–41. doi: 10.1016/j.jped.2013.05.006, PMID: 24156835

[ref18] Chillon P. Hales D. Vaughn A. Gizlice Z. Ni A. Ward D. S. (2014). A cross-sectional study of demographic, environmental and parental barriers to active school travel among children in the United States. Int. J. Behav. Nutr. Phys. Act. 11:61. doi: 10.1186/1479-5868-11-61, PMID: 24885862 PMC4032634

[ref19] Crawford S. B. Bennetts S. K. Hackworth N. J. Green J. Graesser H. Cooklin A. R.. (2017). Worries, 'weirdos', neighborhoods and knowing people: a qualitative study with children and parents regarding children's independent mobility. Health Place 45, 131–139. doi: 10.1016/j.healthplace.2017.03.005, PMID: 28359909

[ref20] D'Haese S. De Meester F. De Bourdeaudhuij I. Deforche B. Cardon G. (2011). Criterion distances and environmental correlates of active commuting to school in children. Int. J. Behav. Nutr. Phys. Act. 8:88. doi: 10.1186/1479-5868-8-88, PMID: 21831276 PMC3168397

[ref21] Dresing T Pehl T. Praxisbuch InterviewTranskription & Analyse. Anleitungen und Regelsysteme für qualitativ Forschende, self-delivered. Marburg (2018).

[ref22] Ecarius J. Handbuch Familie: VS Verlag für Sozialwissenschaften; (2007), Wiesbaden.

[ref23] Faulkner G. E. Richichi V. Buliung R. N. Fusco C. Moola F. (2010). What's “quickest and easiest?” Parental decision making about school trip mode. Int. J. Behav. Nutr. Phys. Act. 7:62. doi: 10.1186/1479-5868-7-62, PMID: 20691063 PMC2924842

[ref24] Forman H. Kerr J. Norman G. J. Saelens B. E. Durant N. H. Harris S. K.. (2008). Reliability and validity of destination-specific barriers to walking and cycling for youth. Prev. Med. 46, 311–316. doi: 10.1016/j.ypmed.2007.12.006, PMID: 18206220

[ref25] Forsberg H. Rutberg S. Mikaelsson K. Lindqvist A. K. (2020). It's about being the good parent: exploring attitudes and beliefs towards active school transportation. Int. J. Circumpolar Health 79, 1–14. doi: 10.1080/22423982.2020.1798113 PMC7480411 32697630

[ref26] Francis J. Martin K. Wood L. Foster S. (2017). ‘I'll be driving you to school for the rest of your life’: a qualitative study of parents' fear of stranger danger. J. Environ. Psychol. 53, 112–120. doi: 10.1016/j.jenvp.2017.07.004

[ref27] Fyhri A. Hjorthol R. Mackett R. L. Fotel T. N. Kytta M. (2011). Children's active travel and independent mobility in four countries: development, social contributing trends and measures. Transp. Policy 18, 703–710. doi: 10.1016/j.tranpol.2011.01.005

[ref28] Gordon-Larsen P. Nelson M. C. Beam K. (2005). Associations among active transportation, physical activity, and weight status in young adults. Obes. Res. 13, 868–875. doi: 10.1038/oby.2005.100 15919840

[ref29] Grize L. Bringolf-Isler B. Martin E. Braun-Fahrlander C. (2010). Trend in active transportation to school among Swiss school children and its associated factors: three cross-sectional surveys 1994, 2000 and 2005. Int. J. Behav. Nutr. Phys. Act. 7:28. doi: 10.1186/1479-5868-7-28, PMID: 20398320 PMC2867987

[ref30] Haug E. Smith O. R. F. Bucksch J. Brindley C. Pavelka J. Hamrik Z.. (2021). 12-year trends in active school transport across four European countries-findings from the health behaviour in school-aged children (HBSC) study. Int. J. Environ. Res. Public Health 18:2118. doi: 10.3390/ijerph18042118, PMID: 33671596 PMC7926861

[ref31] Hipp L. Bünning M. (2020). Parenthood as a driver of increased gender inequality during COVID-19? Exploratory evidence from Germany. European Societies 23, S658–S673. doi: 10.1080/14616696.2020.1833229

[ref32] Hobler D. Klenner C. Pfahl S. Sopp P. Wagner A. (2017). Wer leistet unbezahlte Arbeit? Hausarbeit, Kindererziehung und Pflege im Geschlechtervergleich. Aktuelle Auswertungen aus dem WSI Gender- DatenPortal [WSI Report Nr. 35]. Düsseldorf: Wirtschafts- und Sozialwissenschaftliches Institut (WSI) in der Hans-Böckler-Stiftung.

[ref33] Holloway I. Todres L. (2003). The status of method: flexibility, consistency and coherence. Qual. Res. 3, 345–357. doi: 10.1177/1468794103033004

[ref34] Huertas-Delgado F. J. Herrador-Colmenero M. Villa-González E. Aranda-Balboa M. J. Cáceres M. V. Mandic S.. (2017). Parental perceptions of barriers to active commuting to school in Spanish children and adolescents. Eur. J. Pub. Health 27, ckw249–ckw221. doi: 10.1093/eurpub/ckw249, PMID: 28108594

[ref35] Jacobi D. Caille A. Borys J. M. Lommez A. Couet C. Charles M. A.. (2011). Parent-offspring correlations in pedometer-assessed physical activity. PLoS One 6:e29195. doi: 10.1371/journal.pone.0029195, PMID: 22216207 PMC3247254

[ref36] Johansson K. Laflamme L. Hasselberg M. (2012). Active commuting to and from school among Swedish children-a national and regional study. Eur. J. Pub. Health 22, 209–214. doi: 10.1093/eurpub/ckr042, PMID: 21521708

[ref37] Kremers S. P. de Bruijn G. J. Visscher T. L. van Mechelen W. de Vries N. K. Brug J. (2006). Environmental influences on energy balance-related behaviors: a dual-process view. Int. J. Behav. Nutr. Phys. Act. 3, 1–10. doi: 10.1186/1479-5868-3-9, PMID: 16700907 PMC1481572

[ref38] Larouche R. Saunders T. J. Faulkner G. Colley R. Tremblay M. (2014). Associations between active school transport and physical activity, body composition, and cardiovascular fitness: a systematic review of 68 studies. J. Phys. Act. Health 11, 206–227. doi: 10.1123/jpah.2011-034, PMID: 23250273

[ref39] Lopes F. Cordovil R. Neto C. (2014). Children’s independent mobility in Portugal: effects of urbanization degree and motorized modes of travel. J. Transp. Geogr. 41, 210–219. doi: 10.1016/j.jtrangeo.2014.10.002

[ref40] Loureiro N. Loureiro V. Grao-Cruces A. Martins J. Gaspar de Matos M. (2022). Correlates of active commuting to school among Portuguese adolescents: an ecological model approach. Int. J. Environ. Res. Public Health 19:2733. doi: 10.3390/ijerph19052733, PMID: 35270424 PMC8910768

[ref41] Marzi I. Beck F. Engels E. Renninger D. Demetriou Y. Reimers A. K. (n.d.). Adolescents’ travel behavior in Germany: investigating transport mode choice considering destination, travel distance, and residential setting. Journal of Transport Geography. under review

[ref42] Marzi I. Reimers A. K. (2018). Children's independent mobility: current knowledge, future directions, and public health implications. Int. J. Environ. Res. Public Health 15:2441. doi: 10.3390/ijerph15112441, PMID: 30388880 PMC6267483

[ref43] Mey G Mruck K. Handbuch Qualitative Forschung in der Psychologie. 1st ed. Wiesbaden: VS Verlag für Sozialwissenschaften Wiesbaden; (2010)

[ref44] Nagl-Cupal M. (2013). Theoretical sampling. ProCare 18, 20–22. doi: 10.1007/s00735-013-0213-0

[ref45] Napier M. A. Brown B. B. Werner C. M. Gallimore J. (2011). Walking to school: community design and child and parent barriers. J. Environ. Psychol. 31, 45–51. doi: 10.1016/j.jenvp.2010.04.005

[ref46] Niermann C. Y. N. Gerards S. Kremers S. P. J. (2018). Conceptualizing family influences on Children's energy balance-related behaviors: levels of interacting family environmental subsystems (the LIFES framework). Int. J. Environ. Res. Public Health 15:2714. doi: 10.3390/ijerph15122714, PMID: 30513788 PMC6313966

[ref47] Panter J. R. Jones A. P. van Sluijs E. M. (2008). Environmental determinants of active travel in youth: a review and framework for future research. Int. J. Behav. Nutr. Phys. Act. 5:34. doi: 10.1186/1479-5868-5-34, PMID: 18573196 PMC2483993

[ref48] Pelletier C. A. Cornish K. Sanders C. (2021). Children's independent mobility and physical activity during the COVID-19 pandemic: a qualitative study with families. Int. J. Environ. Res. Public Health 18:4481. doi: 10.3390/ijerph18094481, PMID: 33922530 PMC8122942

[ref49] Physical Activity Parenting Expert Group Mâsse L. C. O’Connor T. M. Tu A. W. Hughes S. O. Beauchamp M. R.. (2017). Conceptualizing physical activity parenting practices using expert informed concept mapping analysis. BMC Public Health 17:574. doi: 10.1186/s12889-017-4487-1, PMID: 28615050 PMC5471850

[ref50] Pucher J. Buehler R. (2008). Making cycling irresistible: lessons from the Netherlands, Denmark and Germany. Transp. Rev. 28, 495–528. doi: 10.1080/01441640701806612

[ref51] Reimers A. K. Marzi I. Beck F. Engels E. Renninger D. Buttazzoni A.. (2022). Active travel behaviour in the family environment: protocol for the mixed-methods cross-sectional ARRIVE study. BMJ Open 12:e056383. doi: 10.1136/bmjopen-2021-056383 PMC8808462 35105596

[ref52] Reimers A. K. Marzi I. Schmidt S. C. E. Niessner C. Oriwol D. Worth A.. (2021). Trends in active commuting to school from 2003 to 2017 among children and adolescents from Germany: the MoMo study. Eur. J. Pub. Health 31, 373–378. doi: 10.1093/eurpub/ckaa141, PMID: 33011779

[ref53] Reimers A. K. Schmidt S. C. E. Demetriou Y. Marzi I. Woll A. (2019). Parental and peer support and modelling in relation to domain-specific physical activity participation in boys and girls from Germany. PLoS One 14:e0223928. doi: 10.1371/journal.pone.0223928, PMID: 31665192 PMC6821055

[ref54] Ross A. Kwon J. Y. Kulinna P. H. Searle M. (2018). Active transportation: the role of parent attitude, the physical environment, and social capital. J. Phys. Act. Health 16, 60–67. doi: 10.1123/jpah.2017-0503 30518299

[ref55] Schoeppe S. Vandelanotte C. Bere E. Lien N. Verloigne M. Kovács É.. (2017). The influence of parental modelling on children's physical activity and screen time: does it differ by gender? Eur. J. Pub. Health 27, 152–157. doi: 10.1093/eurpub/ckw182, PMID: 28177458

[ref56] Smith M. Ikeda E. Duncan S. Maddison R. Hinckson E. Meredith-Jones K.. (2019). Trends and measurement issues for active transportation in New Zealand's physical activity report cards for children and youth. J. Transp. Health 15:100789. doi: 10.1016/j.jth.2019.100789

[ref57] Teixeira P. J. Marques M. M. Silva M. N. Brunet J. Duda J. L. Haerens L.. (2020). A classification of motivation and behavior change techniques used in self-determination theory-based interventions in health contexts. Motiv. Sci. 6, 438–455. doi: 10.1037/mot0000172

[ref58] WHO. Who guidelines on physical activity and sedentary behaviour Geneva: WHO (2020). 33369898

[ref59] Wilson K. Clark A. F. Gilliland J. A. (2018). Understanding child and parent perceptions of barriers influencing children's active school travel. BMC Public Health 18:1053. doi: 10.1186/s12889-018-5874-y, PMID: 30134889 PMC6106832

[ref60] Yang X. L. Telama R. Hirvensalo M. Tammelin T. Viikari J. S. A. Raitakari O. T. (2014). Active commuting from youth to adulthood and as a predictor of physical activity in early midlife: the young Finns study. Prev. Med. 59, 5–11. doi: 10.1016/j.ypmed.2013.10.019, PMID: 24201092

[ref61] Zimmermann J. Tilga H. Bachner J. Demetriou Y. (2021). The effect of teacher autonomy support on leisure-time physical activity via cognitive appraisals and achievement emotions: a mediation analysis based on the control-value theory. Int. J. Environ. Res. Public Health 18:3987. doi: 10.3390/ijerph18083987, PMID: 33920112 PMC8070009

